# ACE2 : S1 RBD Interaction-Targeted Peptides and Small Molecules as Potential COVID-19 Therapeutics

**DOI:** 10.1155/2021/1828792

**Published:** 2021-11-02

**Authors:** Lennox Chitsike, John Krstenansky, Penelope J. Duerksen-Hughes

**Affiliations:** ^1^Department of Basic Sciences, Loma Linda University School of Medicine, 11021 Campus Street, 101 Alumni Hall, Loma Linda, CA 92354, USA; ^2^Department of Pharmaceutical and Applied Life Sciences, Keck Graduate Institute Riggs School of Applied Life Sciences, 535 Watson Dr., Claremont, CA 91711, USA

## Abstract

The COVID-19 pandemic that began in late 2019 continues with new challenges arising due to antigenic drift as well as individuals who cannot or choose not to take the vaccine. There is therefore an urgent need for additional therapies that complement vaccines and approved therapies such as antibodies in the fight to end or slow down the pandemic. SARS-CoV-2 initiates invasion of the human target cell through direct contact between the receptor-binding domain of its Spike protein and its cellular receptor, angiotensin-converting enzyme-2 (ACE2). The ACE2 and S1 RBD interaction, therefore, represents an attractive therapeutic intervention to prevent viral entry and spread. In this study, we developed a proximity-based AlphaScreen™ assay that can be utilized to quickly and efficiently screen for inhibitors that perturb the ACE2 : S1 RBD interaction. We then designed several peptides candidates from motifs in ACE2 and S1 RBD that play critical roles in the interaction, with and without modifications to the native sequences. We also assessed the possibility of reprofiling of candidate small molecules that previously have been shown to interfere with the viral entry of SARS-CoV. Using our optimized AlphaScreen™ assay, we evaluated the activity and specificity of these peptides and small molecules in inhibiting the binding of ACE2 : S1 RBD. This screen identified cepharanthine as a promising candidate for development as a SARS-CoV-2 entry inhibitor.

## 1. Introduction

Since its emergence, COVID-19, the disease caused by the novel SARS-CoV-2, has killed close to 3 million people worldwide and costs world economies trillions of dollars [1, 2]. For nearly a year since COVID-19 was declared a pandemic, the world hoped for a miracle breakthrough in vaccine development. Several vaccines are now available, and the long-awaited miracle has become a source of optimism for billions across the globe. As of now, several prominent vaccines made by Pfizer, Moderna, Johnson & Johnson, AstraZeneca, and others are being administered to people for protective immunity. Many estimates show that over 70% vaccine coverage and uptake is needed in most countries to reach herd immunity, with the US needing to vaccinate about 80–85% of its population [3–6]. In this context, the challenges posed by the emergence of new SARS-CoV-2 variants threaten to derail plans for a vaccine-driven end to the pandemic. New variants such as B.1.351 and P.1 contain mutations in their Spike protein that increase virulence and reinfection rates and confer resistance to antibodies induced by all available vaccines [7–13]. The ongoing SARS-CoV-2 antigenic drift and the fact that many people are not becoming vaccinated due to issues related to vaccine hesitancy, access, or health issues such as an immunocompromised immune system [14–16] suggest that additional COVID-19 therapeutics are needed to fight this pandemic.

Early COVID-19 translational efforts focused on finding therapies for hospitalized patients and led to the discovery and approval of drugs such as dexamethasone that modulate the immune system of critically ill patients [17, 18]. However, for patients with mild-to-moderate COVID-19, monoclonal antibodies (Mabs) that prevent the entry of the virus into cells have been found to be more effective [19, 20]. In addition, emerging studies are demonstrating that Mabs offer prophylactic protection in addition to their therapeutic efficacy [21–23]. This evidence has provided proof of principle for inhibition of viral entry as an effective way to prevent hospitalizations and lessen the risk of developing more fatal complications. Currently, Mab therapies must be administered intravenously at designated infusion centers. Alternative entry inhibitors that are cheaper and could be administered more conveniently are therefore needed for more effective outpatient management of COVID-19. Peptides and small molecules are perfect candidates for therapeutics that are friendly to self-administration in the form of oral pills or inhalants [24, 25]. We designed candidate peptide inhibitors based on the crystal structures of ACE2 and the SARS-CoV-2 Spike protein. For small molecules, we took a repurposing approach with the small molecules to accelerate the development process, since it takes years to develop small molecules into therapeutic drugs *de novo*. Specifically, we focused on peptides and small molecules that were predicted to prevent the interaction of S1 RBD to ACE2. S1 RBD is a domain found in the S1 subunit of SARS-CoV-2 Spike protein that recognizes and binds to the cellular receptor ACE2. The interaction of these two proteins will in turn facilitate the entry of the virus into the cell. The first step of COVID-19 pathogenesis is S1 RBD to ACE2 binding and subsequently virus attachment. Therefore, this is a rational target for therapeutic intervention.

In this study, we developed and optimized an assay based on AlphaScreen™ technology for effectively and quickly screening competitive inhibitors of the S1 RBD : ACE2 interaction. We then designed a set of peptides derived from both sequences of ACE2 and S1 RBD with and without affinity-enhancing or structural stabilization modifications. We also evaluated the potential of small molecules previously reported to have activity against SARS-CoV entry as inhibitors of ACE2 and S1 RBD binding. Our findings show that our assay can be utilized for screening ACE2-targeted entry inhibitors and identified cepharanthine as the most promising candidate of previously identified inhibitors of the S1 RBD : ACE2 interaction.

## 2. Materials and Methods

### 2.1. Reagents

Biotinylated human ACE2 (Cat. no. AC2-H82F9) and His-tagged SARS-CoV-2 S1 RBD (Cat. no. SPD-C52H3) were both purchased from ACROBiosystems (Newark, DE). ACE2 and S1 RBD proteins were diluted into biotin protein buffer (PBS pH 8.0, 5% glycerol, 2 mM DTT) and His protein buffer (20 mM HEPES pH 7.4, 150 mM NaCl, 2 mM KCl, 5% glycerol, 2 mM DTT), respectively. Streptavidin-coated donor beads, nickel chelate acceptor beads, and biotinylated-His peptide were purchased as part of the Histidine detection kit (Cat. no. 6760619C) from Perkin Elmer (Waltham, MA). Emodin (Cat. no. 13109) and cepharanthine (Cat. no. 19648) were purchased from Cayman Chemical Company (Ann Arbor, Michigan). VE607 (Cat. no. 000698941) and SSAA09E2 (Cat. no. AOB4619) were purchased from Molport (Beacon, NY) and Aobious (Gloucester, MA), respectively.

### 2.2. Assay Development and Optimization

The assay for establishing optimal ACE2 and S1 RBD binding was developed by initially cross-titrating the two labeled protein substrates, ACE2 tagged with biotin and S1 RBD tagged with 6xHis based on a protocol adapted from our previously published work [26, 27]. Briefly, 5 *μ*L of each labeled protein was added to wells of a 384-well plate containing 5 *μ*L of blocking buffer solution. The protein substrates were prepared over a 10-point serial dilution ranging from ∼1 nM to 3 *μ*M. The blocking buffer-substrate mix was preincubated for 60 minutes. Streptavidin-coated donor and nickel chelate acceptor beads (20 *µ*g/mL final concentration) were then added. The plates were sealed and incubated for 4 hours at room temperature. The AlphaScreen™ signal was read and quantified using the Perkin Elmer Envision Multilabel plate reader Perkin Elmer Inc. Data analysis was performed and dose-response binding relations were defined using GraphPad Prism plots.

### 2.3. Small-Molecule Inhibitors

To identify small molecule inhibitors that could be used as templates for developing effective entry inhibitors through interference with SARS-CoV-2 binding to ACE2, we searched PubMed using key words “entry inhibitors SARS-CoV” This was further refined by adding other keywords such as “ACE2,” “S1 RBD,” “small molecules,” and “natural products.” From the hits we obtained, we focused on publications that specifically focused on inhibitors that were reported to prevent viral entry and demonstrate competitive antagonism to the binding of S1 of ACE2. From the remaining candidates, we cherry-picked the small molecules with the most citations which were validated in more than one independent experiment.

### 2.4. Peptide Design

We initiated the design process by computationally predicting the residues that contribute the most thermal stability to the binding of ACE2 to S1 RBD based on the crystal complex (PDB ID: 6lzg) and using 2 computational scanning tools: Rosetta and Bude. This approach identified several regions with stabilized interactions, as well as individual residues from these regions that play pivotal binding roles. We comprehensively benchmarked these predicted key determinants against biophysical findings from X-ray-resolved structures available from various recent studies [28–35]. The structural studies reveal that the extended loop subdomain of the S1 RBD, within which some of the key determinants are found, forms a concave pocket that fits the *α*1 helix of the ACE2 peptidase domain (PD). Armed with this knowledge and tools, we extracted the best candidate peptide sequences from the identified stabilized regions. From the ACE2 sequence, peptides with native and nonnative residues were designed. From the native residues, 1 peptide was obtained from the contiguous segment of the *α*1 helix. For peptides containing both native and nonnative residues, we designed 1 peptide from 2 noncontiguous segments, the *α*1 helix and loops 3-4, of the PD linked by a glycine, while the other 3 had mutant residues incorporated in the sequences extracted from the *α*1 helix. Mutant residues were added to either improve binding affinity and/or structural stability in solution. Recent studies have shown that systematic substitution of certain residues in groups 3–7 aa can result in enhanced binding affinity of ACE2 [36, 37]. We incorporated 4–7 mutations into ACE2-based peptides and generated 3 mutant peptides. The other candidate was based on the sequence of S1 RBD. The designed sequences were synthesized and purified by Genescript (>90% yield). We reconstituted the lyophilized peptide powders as per Genescript recommendations.

### 2.5. Inhibitor Dose-Response Relationships

Inhibitor solutions were prepared for a 10-point serial dilution series with the highest final concentration of 200 *μ*M. The final concentration of DMSO was kept below 2% for all molecules tested. Thereafter, the inhibitors were tested for ACE2 : S1 RBD binding inhibition using our optimized AlphaScreen™ protocol. Briefly, 5 *μ*L of 20 nM biotinylated ACE2 and 5 *μ*L of 80 nM of his-tagged S1 RBD were added to wells of a 384-well plate containing 5 *μ*L of blocking buffer solution. 5 *μ*L of each inhibitor (peptide or small molecule) was then added in triplicate at each of the prepared concentration points. The substrate-inhibitor mix was preincubated for 60 minutes at room temperature. Streptavidin-coated donor and nickel chelate acceptor beads (20 *µ*g/mL final concentration) were then added. The plates were sealed and incubated for 4 hours at room temperature. The AlphaScreen™ signal was read and quantified using the Perkin Elmer Envision Multilabel plate reader. Percent inhibition was calculated for each inhibitor relative to the vehicle control, and IC_50_ values were determined from GraphPad Prism dose curves.

### 2.6. Counterscreen Assay

The counterscreen assay was performed using the biotinylated-His_6_ peptide. 10 *µ*L of 5 nM biotinylated-His_6_ peptide substrate was added to plates containing 5 *μ*L of blocking buffer. The peptides and small molecule compounds were prepared using an 11-point serial dilution and then transferred to plates. The mixture was preincubated as described above before adding streptavidin-coated donor and nickel chelate acceptor beads (final concentration 20 *µ*g/mL) and incubating for another 60 minutes. The signals were then read using the Envision™ plate reader. The selectivity index (SI) was calculated from IC_50_ values against ACE2 : S1 RBD as a ratio of the biotinylated-His_6_ peptide.

### 2.7. Data Analysis

Binding and dose-response curves were fitted using GraphPad software (GraphPad Software, Inc., La Jolla, CA).

Signal-to-background ratio was determined as follows:
  (1) $$\begin{array}{c} \frac{S}{B}\text{ ratio}=\frac{{\text{Mean}}_{\text{control}}}{{\text{Mean}}_{\text{background}}}. \end{array}$$
Percent activity and percent inhibition of binding for the compounds were calculated from Alpha Screen signals using the following equations:
  (2) $$\begin{array}{c} \text{Percent activity}:\;100\frac{\left({\text{Mean}}_{\text{compound}}-{\text{Mean}}_{\text{background}}\right)}{\left({\text{Mean}}_{\text{control}}-{\text{Mean}}_{\text{background}}\right)},\\ \text{Percent inhibition}:\;100-\%\text{ activity},\\ \text{Selectivity index}\left(\text{SI}\right):\;\frac{{\text{IC}50}_{\text{Biotin}-\text{His peptide}}}{{\text{IC}50}_{\text{ACE}2-\text{S}1\text{ RBD}}}\geq 10. \end{array}$$

## 3. Results

### 3.1. Assay Development and Optimization

**Figure 1:**
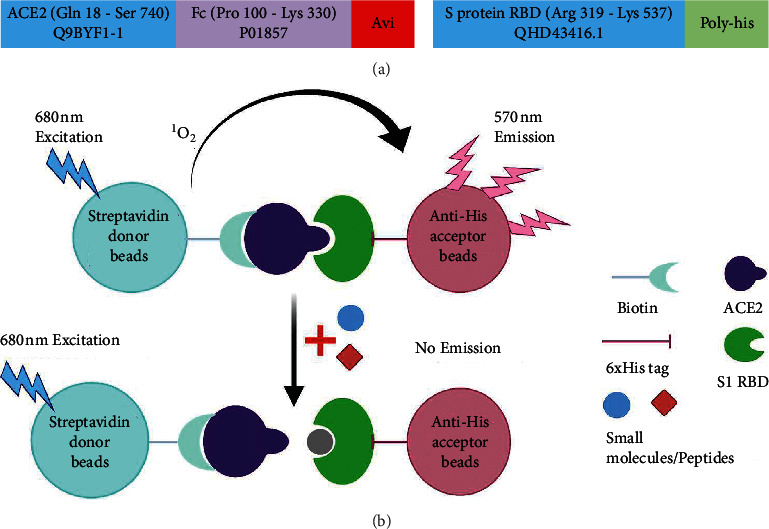
AlphaScreen™-based screening scheme. (a) Human ACE2 tagged with biotin and SARS-CoV-2 S1 RBD tagged with His6 were utilized. The boxes representing the proteins include the residue sequences and the NCBI accession codes. (b) Streptavidin (donor) and nickel chelate (acceptor) beads tether to ACE2 and S1 RBD, respectively. A laser applied at 680 nM excites the donor beads to react with ambient O_2_, activating it to a singlet state (^1^O_2_.) An interaction between the 2 proteins will bring the beads in close proximity (<200 nm) and allow ^1^O_2_ to diffuse and pass on energy to the acceptor bead, causing emission and production of a measurable luminescence signal (570 nm). Small molecules or peptides that disrupt this binding interaction cause signal loss.

**Figure 2:**
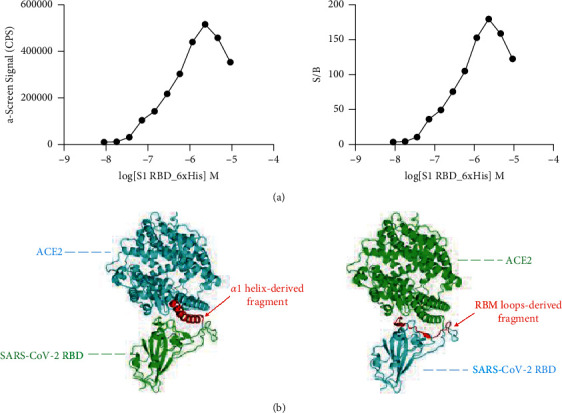
Optimization of assay and design of peptides. (a) Titration of 6xHis-tagged S1 RBD against a fixed concentration of biotin-tagged ACE2 (∼20 nM) (left). Maximal value was obtained at the hook point before diminishing in signal. Signal-to-background (*S*/*B*) ratios at each given concentration used in the titration (right). (b) Interface of the ACE2 and RBD interaction and regions targeted for the rational derivation of peptide fragments (shown in red) that inhibit the binding of the two proteins.

The first goal of our study was to establish a robust assay platform for screening inhibitors of the ACE2 : S1 RBD interaction. To this end, we employed recombinant versions of these proteins labeled with biotin and His tags and developed a proximity assay utilizing luminescent streptavidin and nickel chelate beads as illustrated in Figure 1. To determine the optimal substrate concentrations, assay dynamic range, and sensitivity, we cotitrated biotinylated ACE2 and 6xHis-tagged S1 RBD over a 10-point dilution ranging from 1 nm to 3 *μ*M. Our results show that the two proteins bind to each other, as evidenced by an increase in signal when substrate concentration is increased and the other substrate is held constant. As predicted, with increasing concentrations, titrations reached their highest signal, known as the hook point, after which further increases in substrate concentrations resulted in a diminishment of the AlphaScreen™ signal due to bead saturation (Figure 2(a) and Figure S1). From the assay background emanating from the beads, we were also able to calculate the signal-to-background (*S*/*B*) ratio and thus the assay sensitivity. The *S*/*B* values increased as the concentrations up until the hook point and reached a maximum value of close to 200 (Figure 2(a) and Figure S1). Based on the hook point and S/B values, we chose 20 nM and 80 nM for biotin-ACE2 and 6xHis-S1 RBD, respectively, as the working concentrations for subsequent experiments. These values were chosen because they gave excellent signals and were well below the hook point, thereby avoiding the use of high concentrations that may interfere with bead binding capacity or pairing.

### 3.2. Peptide Inhibitors

**Figure 3:**
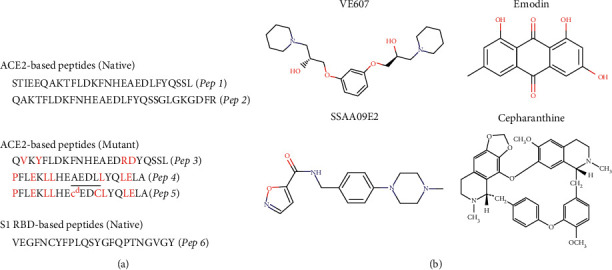
Peptide sequences and small molecule structures. (a) Peptide sequences derived from ACE2 peptidase domain with and without modifications (Pep 1–Pep 5). Pep6 was derived from the S1 RBD sequence. (b) Chemical structures of small molecules VE607, Emodin, SSAA09E2, and cepharanthine. Usually D-amino acids in one letter code is described with lowercase.

The design of the peptides followed 3 simple steps in sequence. First, computational alanine scanning was performed to learn about the predicted per residue energy contribution to the binding energy and the possible hot spot residues. This was followed by benchmarking our computational results with published X-ray crystallography and molecular dynamics simulations data. These results showed that the *α*-1 helix of the peptidase domain (PD) of ACE2 dominates the interface contacts, followed by minor contributions from loop 3-4 and *α*-2 helix. Helix *α*-1 of ACE2 comprises residues 19 to 45, and this stretch of amino acids was predicted to cover about 58% of the interface. We designed our first peptide (Pep 1) based on that stretch (Figure 3). The second contiguous stretch of residues on PD of ACE2 that significantly contributed to the binding comprised residues 351–360, which covered about 30% of the interface. Due to its proximity in space the residues *α*-1 helix, we linked them together through a glycine and created Pep 2 (Figure 3(a)). A similar approach has also been previously utilized by another group [38]. Our computational analysis showed that ACE2 residues at position 19(S), 24(Q), 27(T), 28(F), 30(E), 31(K), 34(H), 35(E), 37(E), 38(D), 41(Y), 42(Q), and 45(L) contributed the most to the binding energy. This information, together with the interface scores of peptide stretches, allowed us to keep these residues mostly intact when we decided to truncate or mutate some residues. We used this knowledge to cut out some residues to maintain a reasonable length when designing Pep 2. To create a super binder peptide (Pep 3) based on mutations empirically found to increase binding affinity, we also kept the aforementioned key residues mostly intact. Additionally, we chose to incorporate 4 amino acid residues found to enhance binding in the study by Chan et al. [36] as shown in Figure 3. Peptides 4 and 5 were designed from the hACE2 (27–46) sequence, replacing residues, not involved in the S1 RBD interaction, with residues that can enhance the *α*-helical conformation for a short peptide in solution. Specifically, Pep 4 has an N-terminal proline to stabilize the peptide to proteases and to help initiate an *α*-helical secondary structure in that it constrains the initial *φ* angle to that consistent with *α*-helical structure. Other substitutions are Glu for Asp and Leu for nonpolar residues in a manner that idealizes the amphipathic *α*-helical pattern in the sequence. These types of modifications have been shown to enhance *α*-helical content in short peptide sequences [39]. Pep 5 additionally includes a covalent side-chain cyclization designed to stabilize the *α*-helical structure [40]. CD studies did not confirm enhancement of a-helical structure for Pep 5 versus Pep 4, but the presence of a D-cysteine in the structure would affect the spectrum and make interpretation difficult using simplified methods of calculation designed for all L-amino acid peptides. Finally, we designed a peptide based on the S1 RBD sequence. Our analysis showed that amino acids of S1 RBD that recognize ACE2 during attachment come from loops 2, 4 and *β*-sheet 6 and they are highlighted by residues such as 484(E), 486(F), 487(N), 493(Q), 498(Q), 501(N), and 505(Y). Therefore, we designed a 23-mer fragment that stretches from 483 to 505. This peptide fragment (Pep 6) was predicted to contribute to about 62% of total interface binding (Figure 3(a)).

### 3.3. Small Molecules

A number of small-molecule candidates were identified from our PubMed search, including emodin, cepharanthine, VE607, SSAA09E2, K22, HTCC, HM-HTCC, MLN-4760, TAPI-0, TAPI-2, sulfamethoxazole, tazobactam, GW280264X, NAAE, hydroxychloroquine, chloroquine, pemirolast, and fidarestat. Some of these hits were associated with binding activity to one or both of the interacting partners. In addition, some candidates were identified in the context of other viruses closely related to SARS-CoV-2 such as SARS-CoV, HCoV-NL63, and HCoV-229E. We cherry-picked the candidates based on the number of citations, connection to SARS-CoV, likely inhibition of the ACE2 : S1 RBD interaction, inhibition of viral entry, and commercial availability. This allowed us to shorten the list to four candidates: emodin, cepharanthine, VE607, and SSAA09E2 (Figure 3(b)).

### 3.4. Competitive Behavior of Peptides and Small Molecules

**Figure 4:**
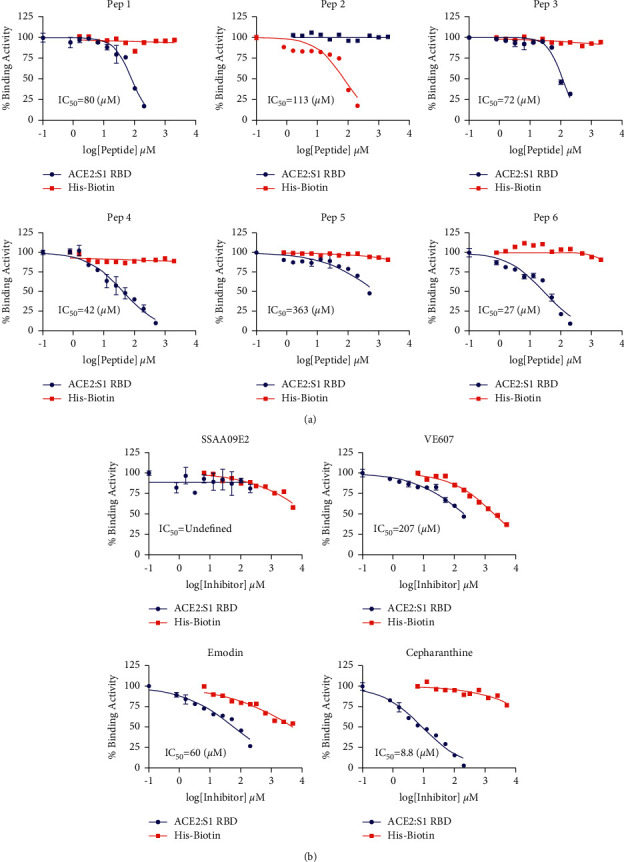
Activity of peptides and small molecules against ACE2 : S1 RBD binding. (a) Dose-response of the peptide candidates (Pep 1–6) against the binding of ACE2 and S1 RBD shown in blue and the respective half-maximal inhibitory concentrations. (b) Dose-response relationships of the 4 small molecules we tested for competitive inhibition of ACE2 : S1 RBD binding.

Using our optimized assay parameters, we collected dose-response data for both the peptides and small molecule candidates. All of the peptides were unlabelled and showed varying degrees of competitive behavior with increases in concentration. Not only did this show that these peptides interfere with the ACE2 : S1 RBD binding, but they also indirectly acted as competition control experiments that show that the assay itself is “inhibitable” (Figure 4(a)). The IC_50_ values of the peptides, except for Pep 5, show that the ACE2-based peptides have similar activities, even though the sequences and in some cases structures, differ significantly. This was particularly true in the case of the hybrid peptide and cyclic peptide. Unexpectedly, the peptides with more extensive modifications were the ones with less activity. Interestingly, the S1 RBD-based peptide showed the highest activity (IC_50_ = 27 *μ*M) in antagonizing the interaction. One potential explanation is that the S1 RBD had a slightly higher interface coverage compared to the native ACE2-based peptide. That is, none of the peptides demonstrated potency stronger than the micromolar range (Figure 4(a)). For the small molecules, there was a wider variation in the effectiveness of the candidate inhibitors. SSAA09E2 showed negligible to zero activity against ACE2 : S1 RBD binding. Emodin and VE607 showed some activity, with intermediate potencies of IC_50_ 60 and 80 *μ*M, respectively. Cepharanthine showed the highest activity of all the inhibitors tested with an IC_50_ below 10 *μ*M (Figure 4(b)). To confirm that the binding behavior from the competition assay was due to specific inhibition of ACE2 : S1 RBD interaction, we employed a counterassay to screen for compounds that interfere with AlphaScreen™ assay conditions rather than protein-protein binding (Figures 4(a) and 4(b)). We used the selectivity index (SI) parameter to determine the specificity of each inhibitor we tested, SI > 10 being the acceptable threshold. The selectivity index results show all peptides had acceptable selectivity profiles (Table 1). Since the peptides were all mostly derived from the native sequences of the proteins being tested, assay nonspecificity was not anticipated. For the small molecules that met the standard, the SI values were 90 for emodin and >200 for cepharanthine. These are good selectivity profiles for small molecules. The SI of SSAA09E2 was not calculable and the SI of VE607 was just below 10. Overall, these data confirm that even though there was wide variation in activity, the majority of the peptides and small molecules we studied here have specific binding inhibition to ACE2 and S1 RBD.

## 4. Discussion

There is a fairly high degree of similarity between SARS-CoV and SARS-CoV-2 in terms of protein sequence identity and binding conformation to ACE2 of their respective RBDs. For this reason, we hypothesized that inhibitors reported as active against the SARS-CoV RBD : ACE2 interaction could serve as initial candidates for antagonizing SARS-CoV-2 entry. Therefore, evaluating these candidates using this knowledge-based approach would serve as an accelerated route towards identifying potential COVID-19 therapeutics as compared to development *de novo*. In this study, we utilized this approach to test this hypothesis and to screen both small molecules and peptides using an S1 RBD : ACE2 binding assay. Despite the traditional perception that peptides suffer from chemical and metabolic liabilities when administered systemically, the fact that COVID-19 is primarily a respiratory disease makes peptides a viable therapeutic through aerosol administration. We also evaluated the inhibitory activity of small molecules with reported SARS-CoV activity.

Overall, our peptides showed modest activity against the S1 RBD and ACE2 interaction. Linear, unmodified peptides based on ACE2 exhibited double-digit potency. The addition of mutant amino acid residues did not affect the peptide activity significantly. The addition of residues that increased helicity also did not significantly improve the activity. Similarly, constraining our peptide fragment did not lead to a significant improvement in peptide activity against S1 RBD : ACE2 binding. Interestingly, the peptide based on S1 RBD had comparably higher activity than most peptides based on ACE2. The results we report in this study come at a time when there are some conflicting results on peptides based on ACE2. Two studies by Karoyan et al. and Curreli et al. have reported that peptides derived from the *α*-1 helix of ACE2 with helical content ranging from 50% to 94% display submicromolar activity against the S1 RBD : ACE2 interaction as well as entry of the virus into the cell [41, 42]. On the other hand, two studies have also reported that peptides extracted from the same region do not have appreciable activity *in vitro* or in cell-based models [43–45]. In one of the studies by Morgan et al., comparable helical contents as high as 72% following stapling did not result in more binding as compared to the native peptide with only 9% helicity [45]. In our case, relatively better results came from the S1 RBD-derived peptide than from ACE2-derived peptides. Nevertheless, the potency was still in the micromolar range. Our results support the published work indicating that peptides have relatively low activity as entry inhibitors and that structural modification may not significantly improve their prospects. It has not escaped our attention that these irregularities in the reported data could be explained by differences in lengths of peptides, techniques, or forms of the virus employed to characterize the interactions in the different studies. Nonetheless, current data suggests that S1 RBD : ACE2-directed peptides may not serve as robust binding inhibitors in their current form. More data is needed to gain additional insights into their potential as COVID-19 therapeutics.

Regarding the small molecules, most of the available evidence regarding anti-CoV activity has been reported in the context of SARS-CoV [46–51]. Specifically, the IC_50_ values reported for cepharanthine, VE607, SSAA09E2, and emodin are 0.98, 3, 3.1, and 200 *μ*M, respectively. Our results varied significantly compared to these numbers. For instance, SSAA09E2 did not even show appreciable activity against S1 RBD : ACE2 binding. Cepharanthine in our hands was 10-fold less active, based on the IC_50_ value we determined. Again, this could be explained by the differences in our methodology and study models compared to those previously used for these small molecules. Consistent with the literature, which reported that cepharanthine displayed one of the lowest IC_50_ values against SARS-CoV-1, we also found cepharanthine to be the most promising compound in our study. The next logical step would be to test its antiviral activity against viral entry into target cells expressing ACE2 using SARS-CoV-2 pseudovirions or WT SARS-CoV-2. Interestingly, the literature shows that several publications have also recently identified cepharanthine from screens using such viral entry assays and ranked it as a promising candidate. Its antiviral activity reported in the literature, depending on the type of virus and target cells used, ranges from 0.73 *μ*M to 30 *μ*M [24, 52–57]. It is, therefore, possible that cepharanthine or more potent derivatives could be developed into entry-blocking therapeutics.

## 5. Conclusions

In summary, we have described an assay that can quickly and easily screen small molecules or biologics in both low and high throughput fashion. This protocol can be used by other labs in screening not only peptides and small molecules, as we have demonstrated, but many other potential entry inhibitors such as miniproteins and nanobodies [58]. Importantly, AlphaScreen™ technology is homogeneous, high-throughput, sensitive, versatile, cost-effective, and together with time-resolved Forster resonance energy transfer (TR-FRET, HTRF), it has been very successful at protein-protein interaction-based drug discovery [59, 60]. We have also partly addressed the question of whether inhibitors that showed activity against SARS-CoV are suitable or viable leads for developing entry inhibitors. The answer is that these small molecules or peptides cannot be expected to serve as COVID-19 therapeutic agents as they currently are configured, given their modest or lacking activity in disrupting S1 RBD : ACE2. Some small molecules such a cepharanthine, however, could potentially serve as templates for building analogue libraries using similarity searches and medicinal chemistry, or developing high-affinity derivatives using structure-guided optimization. Peptides also need to demonstrate improved potency ideally in the nanomolar range or lower, which can be achieved by designing novel peptides or other modifications. Most antibodies that have been studied thus far demonstrate inhibitory concentrations in the nanomolar or subnanomolar ranges, and it is reasonable to expect such high potencies from peptides likely to deliver sufficient efficacy to justify consideration for approval in patients.

## Figures and Tables

**Table 1:** Summary of selectivity indices of peptides and small molecules tested against ACE2 : S1 RBD binding.

Peptide	Selectivity index (SI)	Small molecule	Selectivity index (SI)
Pep 1	>200	SSAA09E2	Not determined
Pep 2	>200	VE607	9.4
Pep 3	>200	Emodin	90
Pep 4	>200	Cepharanthine	>200
Pep 5	>200	N/A	—
Pep 6	>200	N/A	—

## Data Availability

All the data used in this study are included within the manuscript.

## Supplementary Materials

Supplementary Materials Figure S1: titration of biotin-tagged ACE2 against a fixed concentration of 6xHis-tagged S1 RBD (left). Maximal value was obtained at the hook point before diminishing in signal. Signal-to-background (*S*/*B*) ratios at each given concentration used in the titration (right).
