# Supplementary figures and images for: ACE2 : S1 RBD Interaction-Targeted Peptides and Small Molecules as Potential COVID-19 Therapeutics
Source: Adv Pharmacol Pharm Sci. 2021 Nov 2;2021:1828792. doi: 10.1155/2021/1828792 (PMC8564205; doi:10.1155/2021/1828792)

## Slide 1
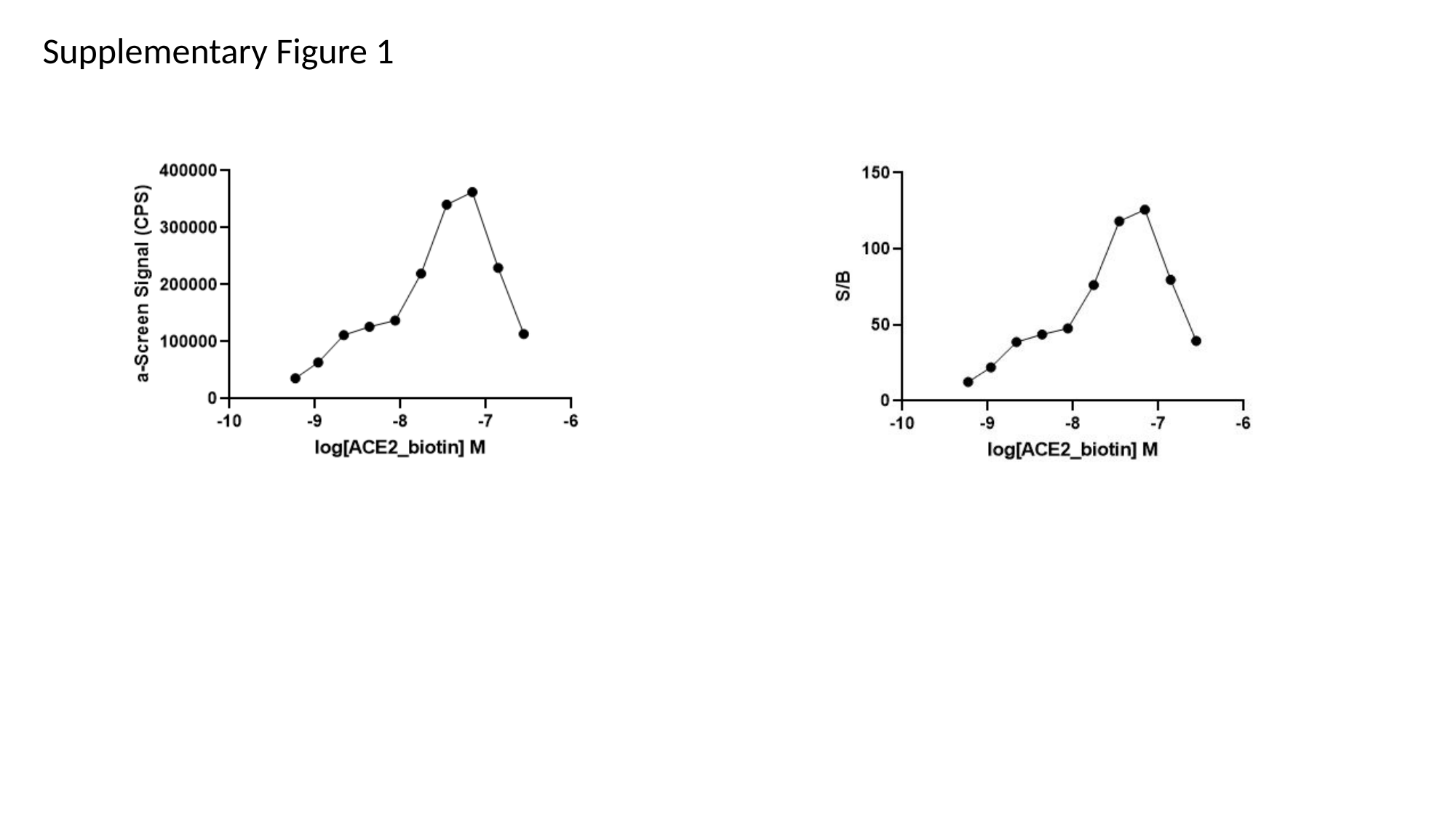

Supplementary Figure 1

Supplement: Supplementary Materials — Figure S1: titration of biotin-tagged ACE2 against a fixed concentration of 6xHis-tagged S1 RBD (left). Maximal value was obtained at the hook point before diminishing in signal. Signal-to-background (S/B) ratios at each given concentration used in the titration (right). [file 1828792.f1.pptx]
